# ATP-Based Ratio Regulation of Glucose and Xylose Improved Succinate Production

**DOI:** 10.1371/journal.pone.0157775

**Published:** 2016-06-17

**Authors:** Fengyu Zhang, Jiaojiao Li, Huaiwei Liu, Quanfeng Liang, Qingsheng Qi

**Affiliations:** Department of State Key Laboratory of Microbial Technology, Shandong University, Jinan, P. R. China; Tsinghua University, CHINA

## Abstract

We previously engineered *E*. *coli* YL104H to efficiently produce succinate from glucose. Furthermore, the present study proved that YL104H could also co-utilize xylose and glucose for succinate production. However, anaerobic succinate accumulation using xylose as the sole carbon source failed, probably because of an insufficient supply of energy. By analyzing the ATP generation under anaerobic conditions in the presence of glucose or xylose, we indicated that succinate production was affected by the intracellular ATP level, which can be simply regulated by the substrate ratio of xylose to glucose. This finding was confirmed by succinate production using an artificial mixture containing different xylose to glucose ratios. Using xylose mother liquor, a waste containing both glucose and xylose derived from xylitol production, a final succinate titer of 61.66 g/L with an overall productivity of 0.95 g/L/h was achieved, indicating that the regulation of the intracellular ATP level may be a useful and efficient strategy for succinate production and can be extended to other anaerobic processes.

## Introduction

Succinic acid, a C_4_-dicarboxylic acid, is a precursor of many important chemicals in the food, chemical, and pharmaceutical industries [1]. Indeed, the widespread importance of succinate has secured its listing among the top 12 chemical building blocks by the U.S. Department of Energy [2]. Industrial succinate producers, such as *Escherichia coli*, *Actinobacillus succinogenes*, *Anaerobiospirillum succiniciproducens*, *Mannheimia succiniciproducens* and *Corynebacterium glutamicum*, constitute a small percentage of all succinate-producing organisms [3]. In the absence of exogenous electron acceptors, *Escherichia coli* accumulates succinate as a minor fermentation product, alongside lactate, formate, acetate and ethanol [4,5].

To improve succinate production by *E*. *coli*, researchers have studied various metabolic engineering strategies [5,6] such as activating the glyoxylate pathway, over-expressing pyruvate-metabolizing enzymes, eliminating competing pathways, and providing reducing equivalents and energy [7–11]. A sufficient supply of reducing equivalents is essential for maximizing the yield of target fermentation products [11]. This need has been demonstrated by providing additional reduced carbohydrates (such as sorbitol) [12] and increasing the *in vivo* NADH availability [10,11,13].

A desired metabolic product can also be enhanced by manipulating the intracellular ATP, which plays a crucial role in many biochemical reactions [14,15]. To increase the production of target metabolites, the ATP supply has been increased, reduced or supplied in multiple phases [16]. In previous studies of succinate production, efforts have been made by increasing the ATP supply. In strains such as *E*. *coli* AFP111, the cell viability during anaerobic succinate production is limited by the lack of sufficient energy for cell growth or maintenance [17]. Insufficient energy supply decreases the succinic acid productivity and enhances the formation of pyruvic and acetic acids. The ATP supply has been increased by recruiting phosphoenolpyruvate (PEP) carboxykinase (PCK) for PEP carboxylation [18–21]. However, ATP is also an allosteric regulator that inhibits the primary enzymes of the glycolytic and pentose phosphate pathways [22]. The decreased pool of ATP relieves the suppression of hexokinase, phosphofructokinase, and glucose-6-phosphate dehydrogenase (G6PDH), increasing the fluxes of these pathways. Inhibition of oxidative phosphorylation has increased the yield of several important industrial microorganisms, and has efficiently improved the production of glycolytic pathway-related products [23–25]. Anaerobic lactate production was also increased by enforcing ATP futile cycling [26].

In the present study, the relationship among ATP generation, sugar ratio in the fermentation medium, and succinate production was explored. Furthermore, the engineered *ptsH*-deleted *E*.*coli* strain was employed to investigate succinate production.

## Materials and Methods

### Bacterial strain and culture medium

The succinate-producing strain YL104H was constructed by our laboratory and its genotype was MG1655 (△*ptsG*, △*poxB*, △*pta*, △*iclR*, △*sdhA*, △*arcA*, △*ldhA*, △*adhE*, *ldhA*::*trc-rbs- glf*_*zm*_, △*ptsH*) [5].

Cells were cultured in Luria–Bertani (LB) medium containing tryptone (10 g/L), yeast extract (5 g/L), and NaCl (10 g/L); or AM1 medium [27] supplemented with varying amounts of glucose and xylose, or with xylose mother liquor derived from biomass (Shandong Longlive Biotechnology Co. Ltd.). The xylose mother liquor contained approximately 300 g/L xylose and 100 g/L glucose, while for succinate production, 50 g/L glucose was added to adjust to a 1:2 glucose: xylose ratio.

### Fermentation

The aerobic–anaerobic dual phase fermentations of glucose, xylose and sugar mixture were carried out in a 1-L fermenter (INFORS HT Multifors, Switzerland). For the first seed culture, a single clone was inoculated in a 300-ml Erlenmeyer flask containing 50 ml LB, and incubated for 12 h with shaking at 250 rpm. Four ml (5% v/v) of the first seed culture was transferred into a 500-ml Erlenmeyer flask containing 80 ml AM1 (with addition of 1 g/L yeast extract and 30 g/L glucose), and shaken at 250 rpm for 22 h, as the second seed culture. All of the second seed culture (10% v/v) was transferred into 800 ml AM1 medium supplemented with a selected carbon source in a 1-L fermenter for batch fermentation. The total fermentation time (70 h) included a 28-h aerobic phase (conveying of air) and a 42-h anaerobic phase (conveying of carbon dioxide). The gas flow rate and agitation were maintained at 1 vvm and 350 rpm, respectively. All cultures were incubated at 37°C. The pH was measured by a glass electrode and controlled at 6.4–6.8 by adding 5 M K_2_CO_3_ and 2 M NaOH.

The aerobic, microaerobic and anaerobic whole-phase fermentations were carried out in a 3-L fermenter (INFORS HT Labfors, Switzerland) containing 2 L AM1 medium, with the sugar mixture or xylose mother liquor as carbon source. The seed culture conditions were those of the dual phase fermentation. The first seed was cultured in a 300-ml Erlenmeyer flask containing 50 ml LB, and ten ml (5% v/v) of the culture was transferred into a 1000-ml Erlenmeyer flask containing 200 ml AM1 (with addition of 1 g/L yeast extract and 30 g/L glucose) as the second seed culture. Then all of the second seed culture (10% v/v) was transferred into the 3-L fermenter containing 2 L AM1 (with addition of 10 g/L ammonium sulfate and the suitable sugar mixture or xylose mother liquor) for succinate production. The aerobic fermentation time was 20 h with the dissolved oxygen increased by 30% (regulated by varying the agitation speed from 300 to 1000 rpm and maintained the air flow rate at 1.5 vvm); the dissolved oxygen was then controlled at 0–1% (regulated by varying the agitation speed from 300 to 1000 rpm, and regulating the air flow rate at 0.5–1.5 vvm) for 30 h for the microaerobic fermentation; finally, the air was replaced with carbon dioxide after 50 h for anaerobic fermentation (maintained the agitation speed and gas flow rate at 350 rpm and 1 vvm). In all fermentations, the temperature and pH were holding at 37°C and 6.4–6.8.

### Fermentation analysis

Biomass was detected by measuring the density at 600 nm (1 OD_600_ ≈ 0.34 g Cell Dry Weight (CDW)/L) with a spectrophotometer (Shimadzu, Japan). The organic acid concentrations were measured by high-performance liquid chromatography (HPLC) (Shimadzu, Japan) and an Aminex HPX-87H ion exclusion column (Bio-Rad, USA). All figures are repeated three times, and their average was shown in the text.

For ATP assays, 4 ml of the bacterial liquid were separated into two samples, one for measuring the cell dry weight after drying at 65°C for 4 h; the other for measuring the intracellular ATP content. Prior to ATP measurements, the cells were broken up in EZLys^TM^ Bacterial Protein Extraction Reagent (BioVision, USA). Proteins in the solution, which would interfere with the measurements, were then eliminated by Deproteinizing Sample Preparation Kit (BioVision, USA). Finally, using the ATP Colorimetric/Fluorometric Assay Kit (BioVision, USA), the ATP concentration was measured by a Microplate Analyzer (BioTek Synergy HT, USA) in fluorometric mode. Final data were obtained as the ATP content divided by the cell dry weight (nmol/g CDW).

### Metabolic model for YL104H and flux balance analysis (FBA)

**Fig 1:**
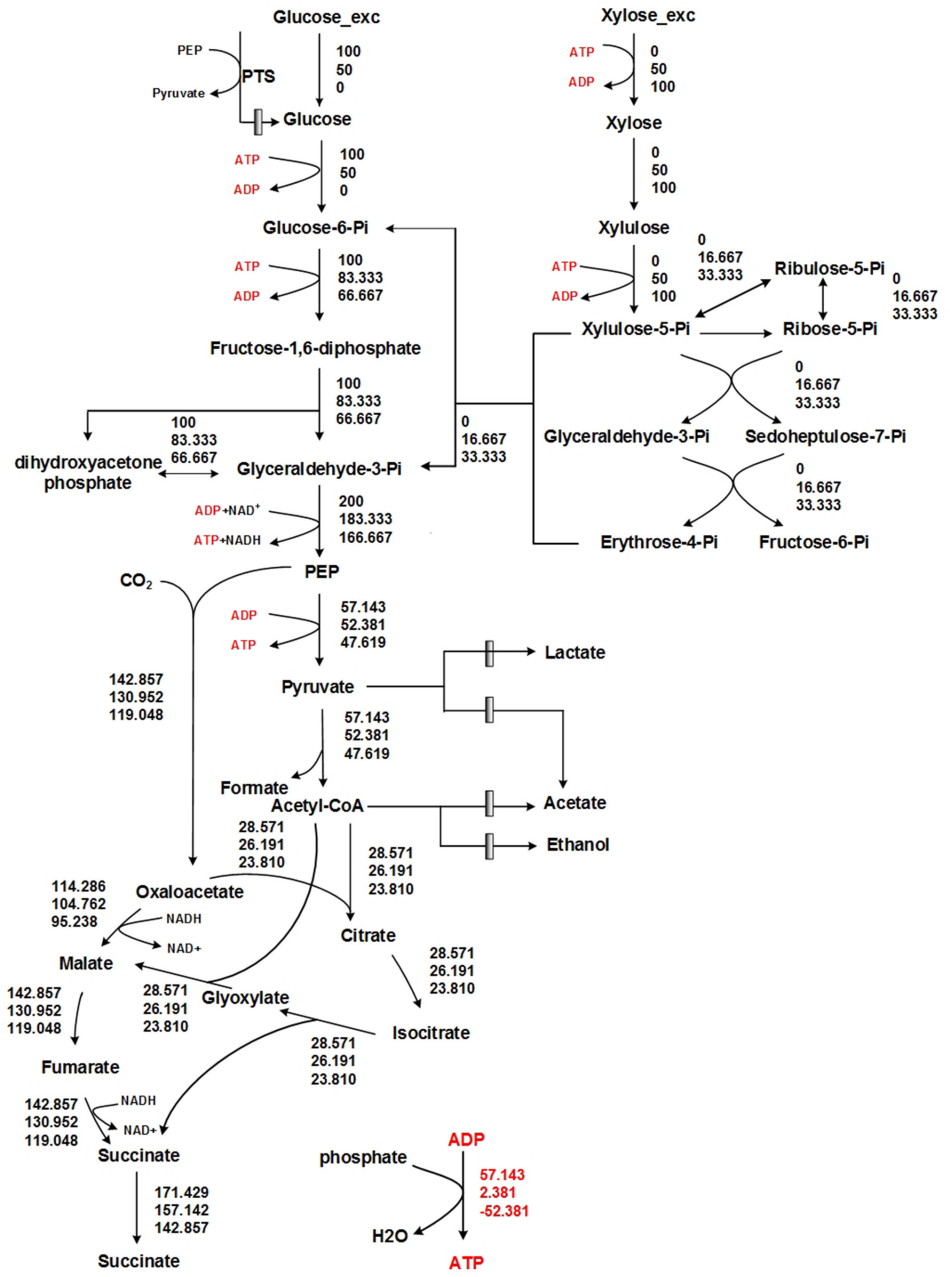
The anaerobic ATP production or consumption on different carbon sources predicted using flux balance analysis (FBA). Solid rectangles denote inactivated pathways, and the metabolic fluxes of reactions were stated alongside. The first, second and third row of numbers corresponding to glucose, mixed glucose-xylose (1:1) and xylose.

The metabolic model for YL104H and flux balance analysis (FBA) method was applied for analyzing the ATP production or consumption on different carbon sources. During anaerobic culture, *E*. *coli* undergoes mixed-acid fermentation that yields acetate, ethanol, formic acid, and lactate as its major fermentation products. Therefore, except formic acid, the formation of other by-products were suppressed in YL104 [4]. An isolated metabolic subnet model that composed by 41 reactions was constructed and used for all simulations (EcoliGX01) (Fig 1 and S1 Table) [4]. The information about reversibility or irreversibility of included reactions was extracted from KEGG database (http://www.kegg.com) except the ATP-ADP exchange reaction was manually set for calculation convenience. As the main constrained condition, neither uptake nor secretion of coenzyme (NAD, NADH) was allowed. All simulations shared an identical objective function: maximizing the succinate production under a limiting carbon source uptake, and all simulations were carried our using the RAVEN Toolbox in the MATLAB environment [28].

## Results and Discussion

### Succinate production on glucose, xylose or sugar mixture substrate

Glucose and xylose constitute over 90% of the total sugars in lignocellulosic hydrolysates [21,29,30], of which the relative proportion varies widely among different sources [31]. However, co-utilization of both sugars by *E*. *coli* is inhibited by carbon catabolite repression (CCR) [32]. CCR unequivocally presents the first barrier to the production of lignocellulosic fuels and chemicals by microbial fermentation. Deletion of *ptsG*, encoding an EIICB protein in the glucose-specific phosphotransferase system (PTS), induces the co-utilization of xylose and glucose [4]. In our previous study, the Δ*ptsH* mutant strain YL104H showed a different substrate phenotype and accumulated more succinate from glucose than the parent Δ*ptsG* mutant strain YL104 [5].

In this study, we first evaluated the succinate production of YL104H in the presence of glucose, xylose and a sugar mixture (comprising glucose and xylose at identical mass concentrations). The dual-phase fermentation technology was employed (Table 1 and S1 Fig). Under aerobic conditions, YL104H accumulated 39% more succinate in the sugar mixture than in glucose alone. The total sugar utilization was also higher. When cells co-utilized xylose and glucose, the utilization of xylose can provide carbon for cell growth and regulate energy in vivo metabolism. So the cells perform better when grown on the sugar mixture rather than glucose alone [5]. Under anaerobic conditions, the succinate accumulations in the presence of glucose, xylose and mixed sugar substrates were 12.74 g/L, 3.10 g/L and 12.61 g/L, respectively. Interestingly, almost no anaerobic succinate accumulated in the presence of xylose-only substrate, indicating that a limiting factor exists under anaerobic fermentation. Overall, the highest succinate production by YL104H (30.67 g/L) was achieved in the sugar mixture after 60 h cultivation.

**Table 1:** Dual-phase fermentation of YL104H in glucose, xylose, or mixed glucose–xylose.

Carbon source	Biomass (g CDW/L)	Glucose consumption (g/L)		Xylose consumption (g/L)		Succinate production (g/L)		Overall yield (mol/mol)	Overall productivity(g/L/h)
		aerobic	anaerobic	aerobic	anaerobic	aerobic	anaerobic		
**Glucose**	**2.86±0.10**	**18.26±1.34**	**11.35±1.83**			**12.99±1.83**	**12.74±0.73**	**1.32±0.13**	**0.37±0.07**
**Xylose**	**2.01±0.10**			**19.13±3.11**	**5.27±1.56**	**11.69±0.67**	**3.10±0.58**	**0.78±0.15**	**0.21±0.06**
**The sugar mixture**	**2.45±0.25**	**13.68±1.21**	**6.85±0.53**	**15.05±1.15**	**6.00±1.24**	**18.06±0.24**	**12.61±0.36**	**1.03±0.14**	**0.44±0.08**

All cultivations were performed at 37°C in a 1 L fermenter containing AM1 medium for 70 hours (28 h aerobic phase; 42 h anaerobic phase). The carbon sources of the three groups were 40 g/L glucose, 40 g/L xylose, as well as 30 g/L glucose and 30 g/L xylose, respectively. Overall yield and productivity were calculated by the aerobic and anaerobic succinate production divided by the number of aerobic and anaerobic sugar consumption moles or the overall fermentation time.

### Analysis of ATP level during the dual-phase fermentation

When xylose was the sole carbon source, YL104H accumulated almost no succinate under anaerobic conditions (Table 1 and S1 Fig). We attribute this inhibition to insufficient ATP supply during the anaerobic fermentation of xylose. To support this inference, Flux balance analysis (FBA) was performed to investigate the ATP production or consumption on different carbon sources as described in Materials and methods and results are shown in Fig 1, and S1 Table. The fluxes are based on 100 moles of sugars uptake. The net ATP generation is negative predicted by the model when xylose is the sole substrate of succinate production (Fig 1). During anaerobic fermentation, the conversion of xylose to pyruvate yields 0.67 net ATPs per xylose, as one ATP is required for each of xylose transport and xylulose phosphorylation [33]. Although the generation of acetate from pyruvate generates one ATP [19,34], this capability is lost in YL104H, which lacks a functional acetate secretion pathway. The carbon flux distributed between the reductive TCA cycle and the glyoxylate pathway at the PEP node with a weighting of 5:2 (Fig 1). In this situation, only 28.6% of PEP react to pyruvate and generate ATP, the net yield of ATP per xylose to succinate in *E*. *coli* YL104H was produced as -0.524 mol/mol, while the net yield of ATP per glucose and a sugar mixture (glucose: xylose = 1:1) to succinate was 0.571 mol/mol and 0.024 mol/mol respectively, predicted by the model (Fig 1). Additionally, the succinate yield from glucose, xylose and mixed sugar were predicted by the model in the situation without considering ATP availability (Fig 1).

**Fig 2:**
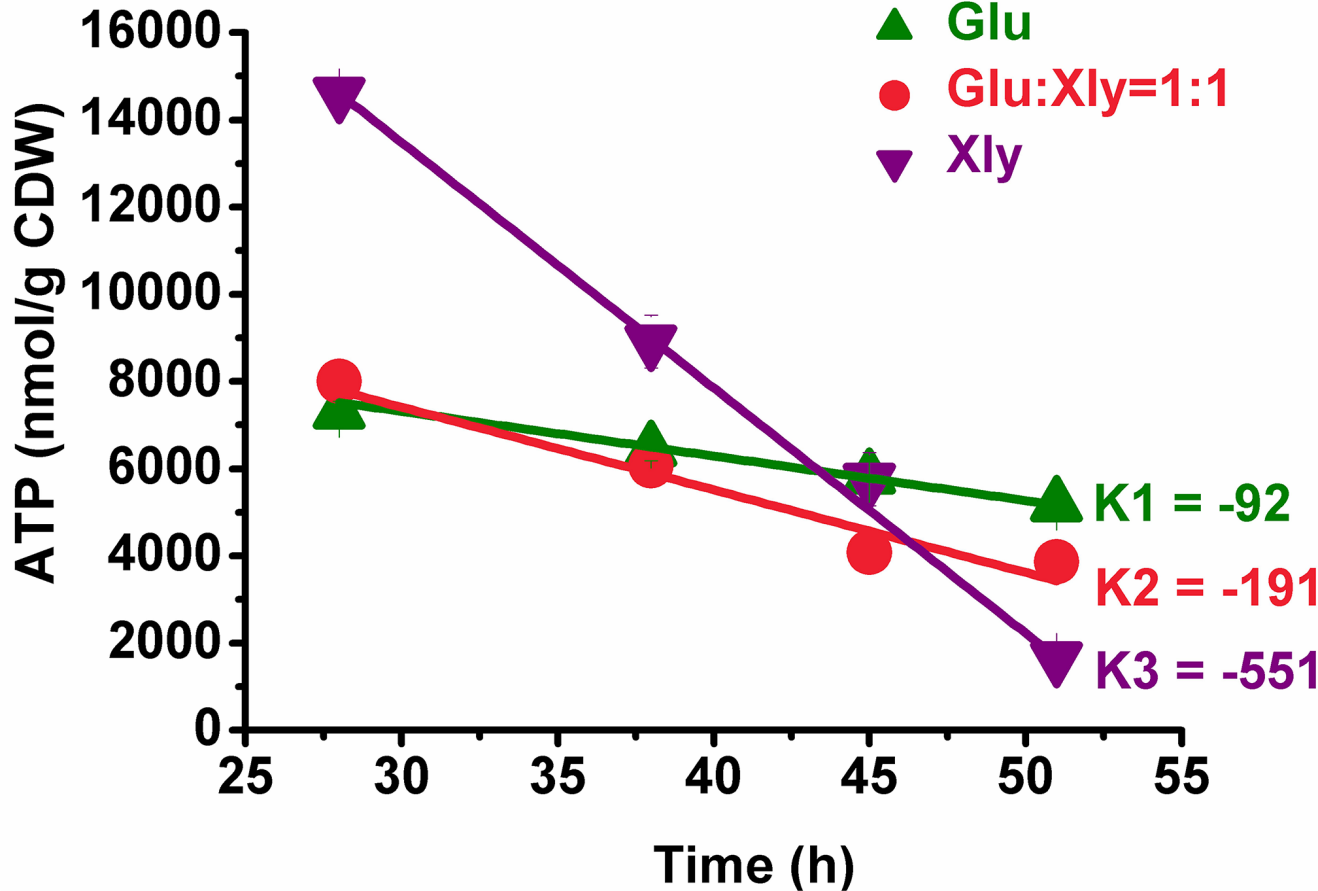
Intracellular ATP levels in the presence of different carbon sources. Data are the ATP contents per cell dry weight, and the regression lines were calculated by Origin Pro 8.0. All slopes (*k*) of these regression lines were listed beside, and their regression coefficients were all above 0.95. All the date were measuring at the anaerobic fermentation stage between 28 to 70 hours.

Because YL104H cannot grow anaerobically, the above FBA results were verified by measuring the ATP generated during the aerobic–anaerobic dual-phase fermentation process. The anaerobic ATP level was significantly affected by the generated aerobic ATP, which had supposedly accumulated for anaerobic consumption [35]. Regardless, the anaerobic ATP content should be significantly reduced from its aerobic levels. Thus, the tendency of the regression line (the slope *k*), which was calculated by ATP level throughout the whole fermentation process and represented the specific rate of ATP consumption in units of nmol/(g CDW)/h, is important [36]. In the present study, the slopes *k*1 for glucose fermentation and *k*3 for xylose fermentation were -92 and -551, respectively (see Fig 2), indicating that anaerobic xylose fermentation generated less ATP than anaerobic glucose fermentation. The slope *k*2 (-191) in mixed-sugar substrate was intermediate between *k3* and *k1* (Fig 2), indicating that the ATP derived from glucose fermentation may complement xylose fermentation during anaerobic succinate production.

### Regulation of ATP generation by optimizing the sugar ratios for improved succinate production

In the aforementioned experiment, xylose can facilitate the co-utilization of aerobic sugars and succinate accumulation in YL104H. However, the negative ATP generation affected succinate production from xylose in YL104H under anaerobic condition. Therefore, we varied the ratio of glucose to xylose (3:1, 2:1, 1:2 and 1:3) and compared the succinate production by YL104H. As shown in Table 2 and S2 Fig, the highest succinate titer was 36.26 g/L (at a glucose-to-xylose mass ratio of 1:2). At glucose-to-xylose mass ratios of 3:1, 2:1 and 1:3, the final succinate titers were 26.63 g/L, 30.47 g/L and 21.56 g/L, respectively.

**Table 2:** Dual-phase fermentation of YL104H for different glucose-to-xylose mass ratios.

Ratio of glucose to xylose	Biomass (g CDW/L)	Glucose consumption (g/L)	Xylose consumption (g/L)	Succinate production (g/L)	Overall yield (mol/mol)	Overall productivity (g/L/h)
**3:1**	**2.47±0.27**	**17.70±1.49**	**9.81±0.97**	**26.63±1.38**	**1.39±0.09**	**0.38±0.04**
**2:1**	**2.54±0.45**	**18.45±1.79**	**15.03±1.21**	**30.47±1.24**	**1.28±0.07**	**0.44±0.02**
**1:2**	**2.61±0.20**	**13.58±1.65**	**26.21±1.83**	**36.26±2.04**	**1.24±0.07**	**0.52±0.03**
**1:3**	**2.18±0.33**	**9.77±1.00**	**17.44±1.87**	**21.56±1.65**	**1.08±0.06**	**0.31±0.03**

The fermentation conditions were the same as the conditions of Table 1. And the carbon sources of the four groups were 33 g/L glucose and 11 g/L xylose, 30 g/L glucose and 15 g/L xylose, 15 g/L glucose and 30 g/L xylose, as well as 11 g/L glucose and 33 g/L xylose, respectively. During the fermentation, the carbon source was supplemented proportionally to ensure the sugar content higher than 5 g/L.

**Fig 3:**
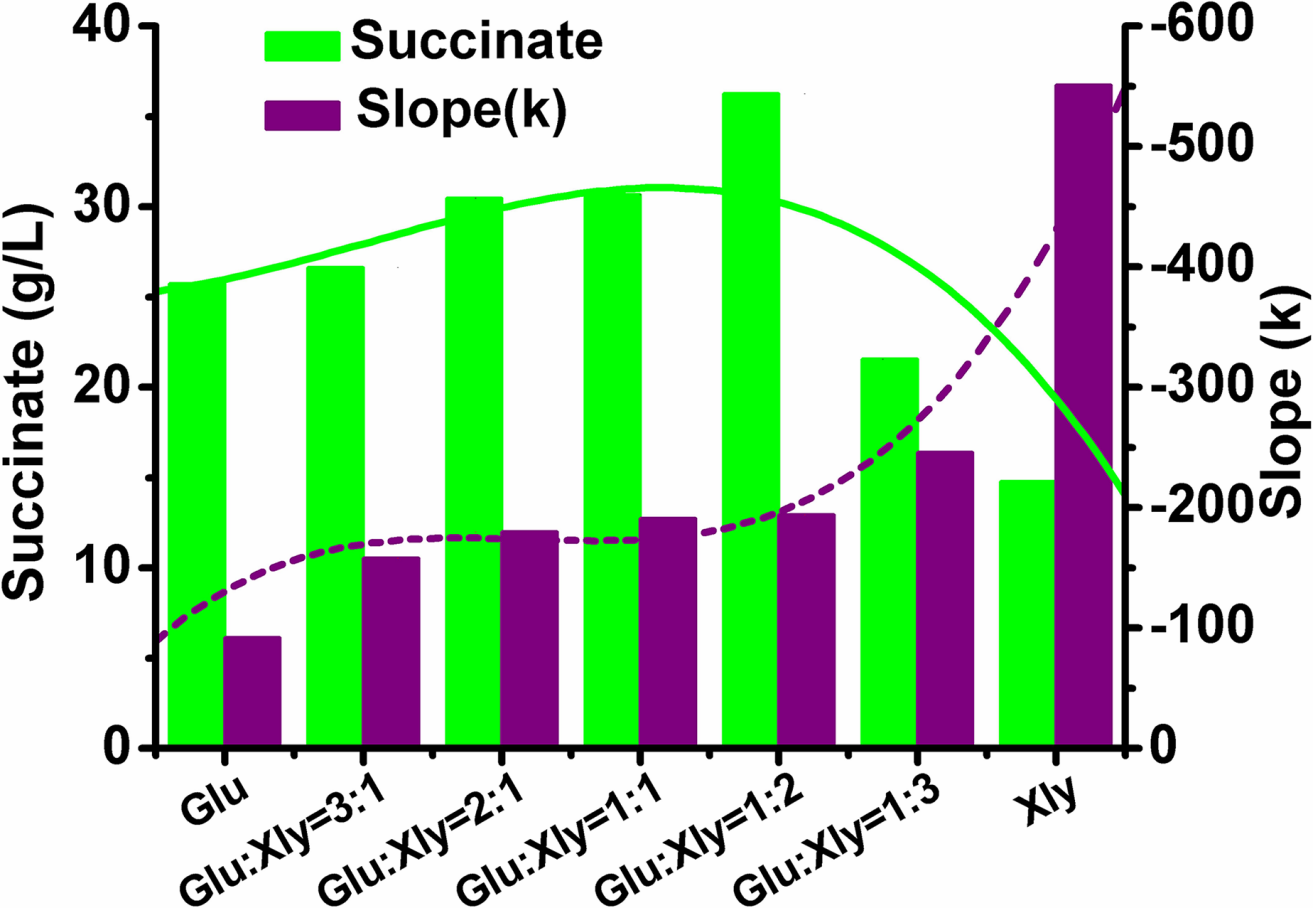
Relationship among succinate production, sugar ratio and intracellular ATP level. The ATP level was shown as the slops of their ATP regression lines which represented the specific rate of ATP consumption in units of nmol/(g CDW)/h. The solid and dashed lines were the second order polynomial regression line calculated by Origin Pro 8.0.

The regression curves of ATP levels throughout the fermentation process reveal a relationship among the sugar ratio, ATP generation and succinate production (Fig 3). The regression slopes of the ATP level versus time plots decreased with increasing proportion of xylose in the sugar mixture. This is in accordance with our aforementioned FBA results. On the contrary, the succinate titer increased up to a glucose-to-xylose ratio of 1:2. Thereafter, the succinate accumulation decreased and the regression slope increased. These results showed that the ratio of glucose to xylose in the substrate affects the intracellular ATP generation; consequently, the intracellular ATP generation may be adjusted by regulation of glucose and xylose ratio to improve the succinate production. Additionally, YL104H cannot grow anaerobically. Therefore, all of ATP was almost utilized to accumulate succinate (S1 Fig).

ATP plays a vital role in cell growth and the production of target metabolites [37]. Under anaerobic conditions, ATP generation is positively related to cell growth and succinate accumulation [18]. Therefore, coupling the ATP generation with cell growth improves the anaerobic succinate accumulation from glucose. However, anaerobic fermentation generates less ATP from xylose than from glucose substrate. To improve the anaerobic ATP supply from xylose, researchers have introduced a carboxykinase (PCK) enzyme that generates ATP through pyruvate conversion [19–21].

YL104H grew aerobically during the dual-phase fermentation, but cell growth ceased when the cultivation conditions were altered to anaerobic, indicating that ATP and NADH consumption was also terminated (S1 Fig). Such growth cessation would further affect the substrate uptake. Phosphofructokinase, which controls the metabolic flux through the glycolytic pathway, is inhibited by high ATP concentrations and high NADH/NAD^+^ ratios [22]. Decreased ATP generation facilitates carbon flux through the glycolytic pathway [38] and probably improves the succinate accumulation. Therefore, the anaerobic ATP level should be maintained at levels that enhance the succinate production. Regulating the ATP generation by adjusting the glucose-to-xylose ratio in the substrate probably provides a simple and efficient method for improving succinate production.

### Validation of succinate production from biomass in a 3-L bioreactor

**Fig 4:**
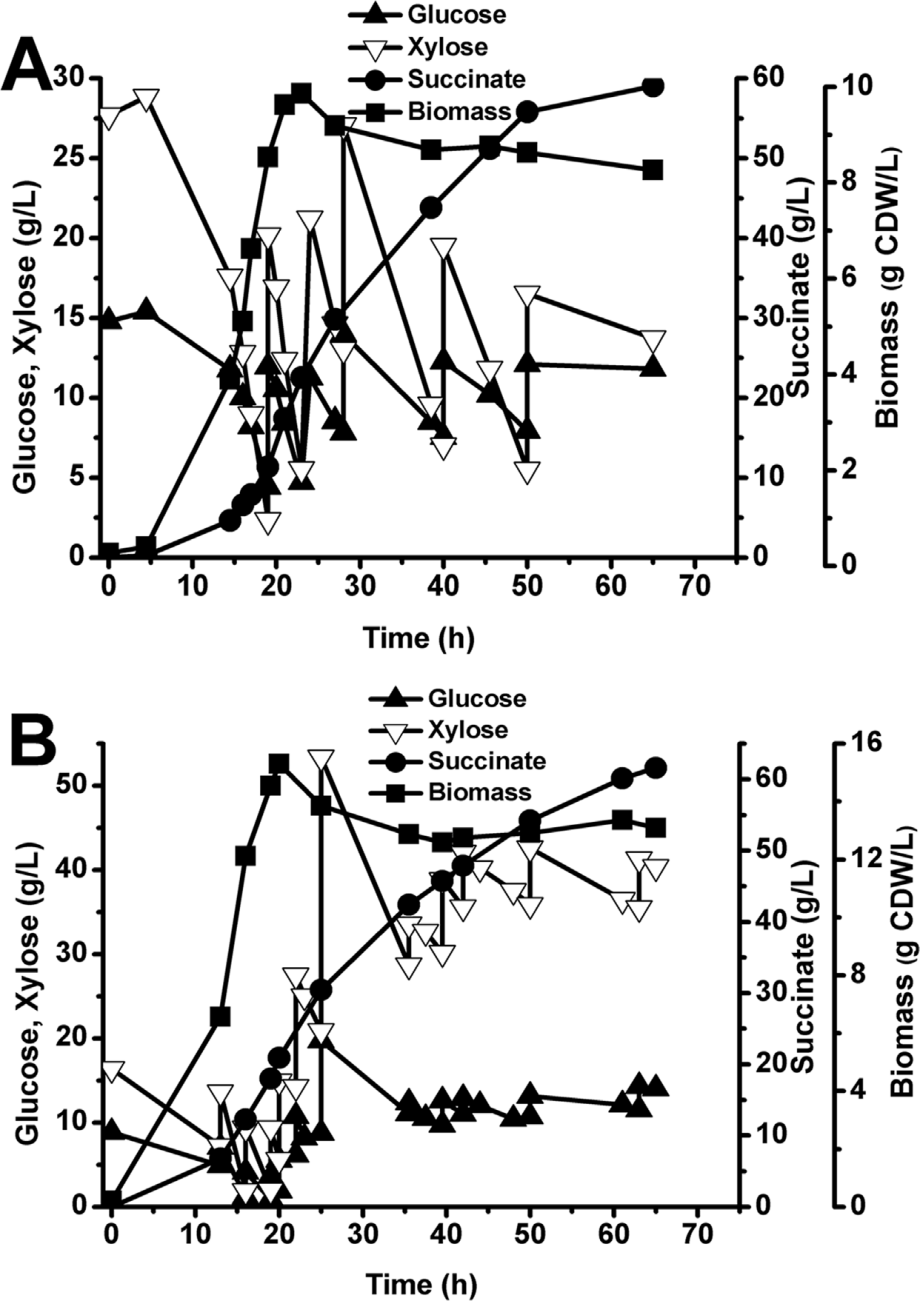
Succinate production in a 3-L bioreactor. Succinate production, sugar consumption and biomass of YL104H in artificial sugar mixture (A) and xylose mother liquor (B) under aerobic, microaerobic and anaerobic whole-phase fermentations. The xylose mother liquor contained approximately 300 g/L xylose and 100 g/L glucose, and 50 g/L glucose was added to adjust the glucose: xylose ratio at 1:2.

To validate the succinate production in mixed-sugar substrate in the 3-L fermenter, an artificial mixture with a glucose-to-xylose ratio of 1:2 was prepared. In this mixture, the fermentative succinate titer of *E*. *coli* YL104H was 59.02 g/L, and the overall volumetric productivity was 0.91 g/L/h (Fig 4A). Succinate production from xylose and glucose as carbon sources has been attempted in several studies. To improve the succinate yields, researchers have inactivated *ptsG* [12,39], pyruvate formate lyase (PFL) and lactate dehydrogenase (LDH) [11]. Based on above studies, the resulting strain AFP184, which lacks functional PFL and LDH enzymes, can ferment both five- and six-carbon sugars and exhibits strong growth characteristics [17]. To improve the efficiency of succinic acid production during glucose and xylose fermentation of strains derived from the parental strain K12, Jiang’s group also deleted the *ptsG* gene. Hydrolysate fermentation by the engineered strain under anaerobic conditions generated 39.3 g/L of succinic acid [20]. This study demonstrates that deletion of *ptsH* is an effective strategy for the improvement of succinate production in mixed glucose–xylose substrate.

To determine whether this result is achievable in actual biomass, the strain was cultivated in xylose mother liquor, an acid-hydrolysis by-product of xylose production from sugarcane bagasse or corncob, which is widely available in China and other countries [40]. The market price of xylose mother liquor is approximately 125 euros/ton sugar, around one-third the price of corn-based sugar. Therefore, xylose mother liquor shows great promise as an alternative fermentation feedstock [41]. In xylose mother liquor, YL104H generated 61.66 g/L succinate after 65 h fermentation, with an overall volumetric productivity of 0.95 g/L/h (Fig 4B), higher than in the artificial sugar mixture. Therefore, succinate was efficiently produced from biomass, and a high succinate titer was achieved from the biomass substrate.

As the most abundant biomass source, lignocellulose is being increasingly considered as an alternative feedstock in bioindustries. To boost succinate production from low-cost sugar feedstock, researchers have engineered various *E*. *coli* strains [17,19,42,43]. However, most of prior studies have used complex medium supplements such as tryptone, yeast extract, corn steep liquor, or Luria broth. In minimal medium containing glucose and xylose, an engineered *E*.*coli* BA305 accumulated 23.1 g/L succinate with a productivity of 0.24 g/L/h [43]. Very recently, Sawisit et al. engineered the succinate-producing *E*. *coli* strain AS1600a, which contains a novel *galP* mutation. This strain produced 72.7 g/L succinate from lignocellulose in 144 h with a productivity of 0.50 g/L/h [44]. In this study, YL104H produced 61.66 g/L succinate with a productivity of 0.95 g/L/h in minimal medium containing xylose mother liquor as the carbon source. Adjusting the glucose-to-xylose ratio in the substrate simply and effectively improves the succinate production by regulating the intracellular ATP generation. In addition, we recently proved that deleting various elements of the PTS system in *E*. *coli* alters the utilization of sugar mixtures supplied at various glucose-to-xylose ratios [22]. This indicates that succinate-producing strains can be tailored to different biomasses.

## Conclusions

The present study demonstrated for the first time that intracellular ATP generation can be regulated by the glucose and xylose ratios in the substrate, and that succinate production might be optimized by regulating the intracellular ATP generation. Furthermore, 61.66 g/L succinate with an overall volumetric productivity of 0.95 g/L/h was achieved in xylose mother liquor derived from biomass. Therefore, the present study indicates that the regulation of the intracellular ATP level is may be useful and efficient strategy for succinate production and may be extended to other anaerobic processes.

## Supporting Information

S1 Fig **Dual-phase fermentation of YL104H in different carbon sources:** (A) glucose; (B) xylose; (C) equal proportions of glucose and xylose. All cultivations were performed at 37°C in a 1 L fermenter containing AM1 medium. The total fermentation time was 70 h (28 h aerobic phase; 42 h anaerobic phase).
(TIF)

S2 Fig **Succinate yields, glucose and xylose consumptions, and cell dry weights at different mass concentration ratios of glucose to xylose:** (A) 3:1, (B) 2:1, (C) 1:2, and (D) 1:3.
(TIF)

S1 Table The isolated metabolic subnet model of YL104H. The information about reversibility or irreversibility of included reactions was extracted from KEGG database (http://www.kegg.com).
(DOCX)
