# Supplementary figures and images for: ATP-Based Ratio Regulation of Glucose and Xylose Improved Succinate Production
Source: PLoS One. 2016 Jun 17;11(6):e0157775. doi: 10.1371/journal.pone.0157775 (PMC4912068; doi:10.1371/journal.pone.0157775)

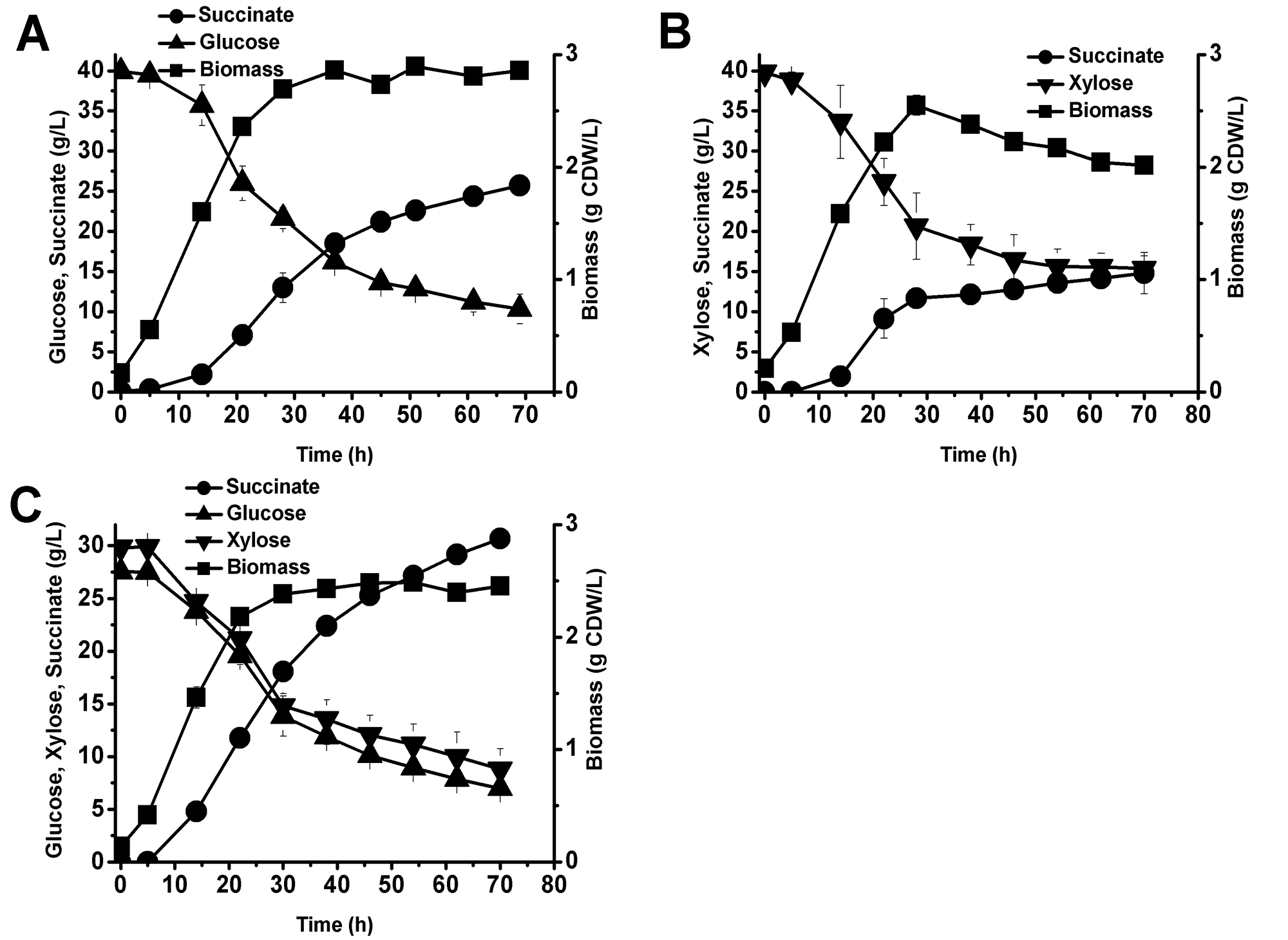

Supplement: S1 Fig — Dual-phase fermentation of YL104H in different carbon sources: (A) glucose; (B) xylose; (C) equal proportions of glucose and xylose. All cultivations were performed at 37°C in a 1 L fermenter containing AM1 medium. The total fermentation time was 70 h (28 h aerobic phase; 42 h anaerobic phase). (TIF) [file pone.0157775.s001.tif]

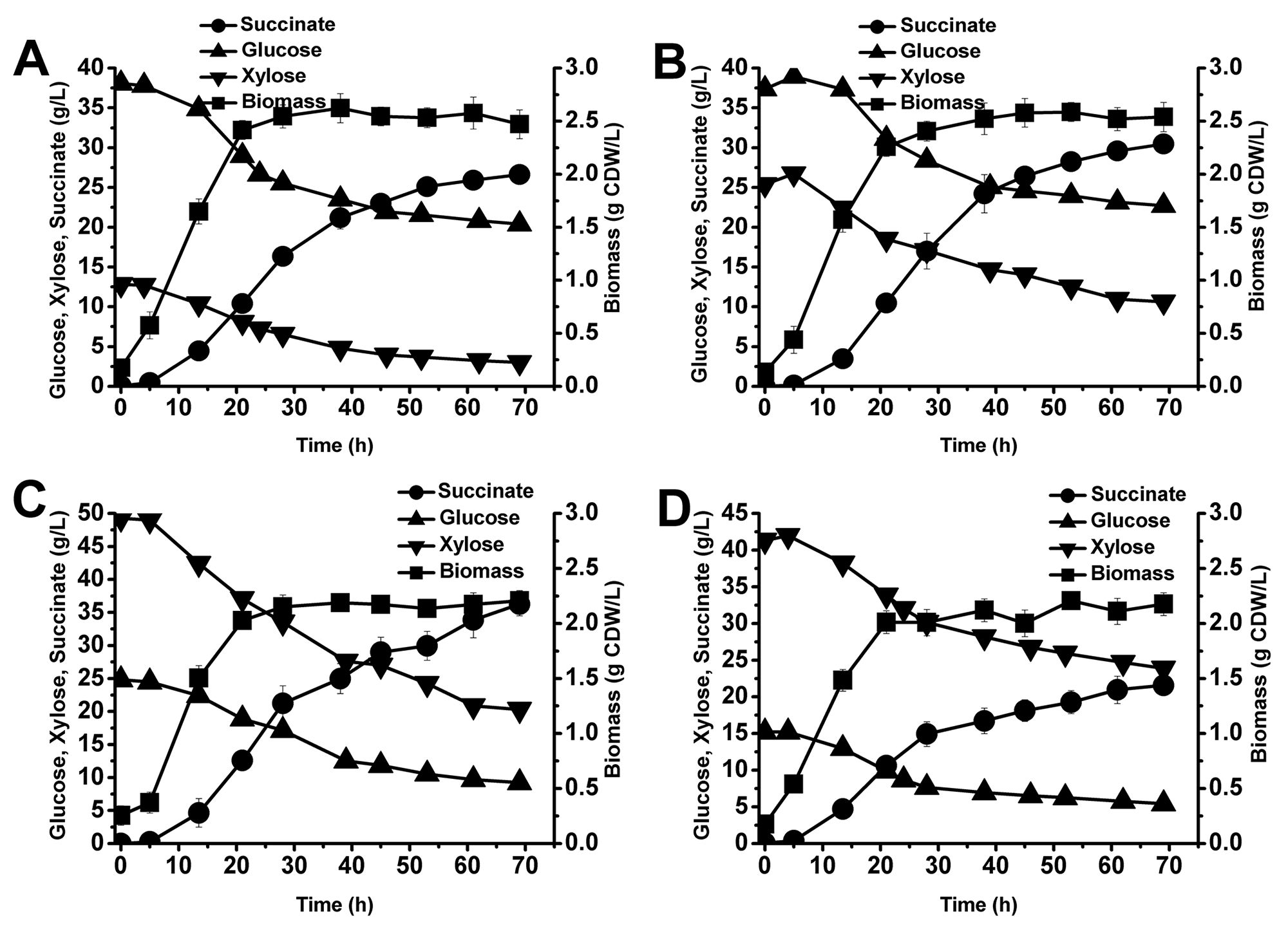

Supplement: S2 Fig — Succinate yields, glucose and xylose consumptions, and cell dry weights at different mass concentration ratios of glucose to xylose: (A) 3:1, (B) 2:1, (C) 1:2, and (D) 1:3. (TIF) [file pone.0157775.s002.tif]
