# Supplementary material for: ATP-Based Ratio Regulation of Glucose and Xylose Improved Succinate Production
Source: PLoS One. 2016 Jun 17;11(6):e0157775. doi: 10.1371/journal.pone.0157775 (PMC4912068; doi:10.1371/journal.pone.0157775)
Supplement: S1 Table — The information about reversibility or irreversibility of included reactions was extracted from KEGG database (http://www.kegg.com). (DOCX) [file pone.0157775.s003.docx]

| IDB1:D34 | NAME | EQUATION |
| --- | --- | --- |
| r1 | transport of D-xylose | D-xylose[e] => D-xylose[c] |
| r2 | transport of glucose | glucose[e] => glucose[c] |
| r3 | D-xylose to D-xylulose | D-xylose[c] + ATP[c] => D-xylulose[c] + ADP[c] + phosphate[c] |
| r4 | hexokinase | glucose[c] + ATP[c] => glucose 6-phosphate[c] + ADP[c] |
| r5 | D-xylulose to D-xylulose-5p | D-xylulose[c] + ATP[c] => xylulose 5-phosphate[c] + ADP[c] |
| r6 | phosphoglucose isomerase | glucose 6-phosphate[c] <=> fructose 6-phosphate[c] |
| r7 | phosphofructokinase | fructose 6-phosphate[c] + ATP[c] => fructose 1,6-bisphosphate[c] + ADP[c] |
| r8 | fructose bisphosphate aldolase | fructose 1,6-bisphosphate[c] => glyceraldehyde 3-phosphate[c] + dihydroxyacetone phosphate[c] |
| r9 | triosephosphate isomerase | dihydroxyacetone phosphate[c] => glyceraldehyde 3-phosphate[c] |
| r10 | glyceraldehyde phosphate dehydrogenase | glyceraldehyde 3-phosphate[c] + phosphate[c] + NAD[c] => 1,3-bisphosphoglycerate[c] + NADH[c] |
| r11 | phosphoglycerate kinase, phosphoglycerate mutase and enolase | 1,3-bisphosphoglycerate[c] + ADP[c] => PEP[c] + ATP[c] + H_2_O[c] |
| r12 | pyruvate kinase | PEP[c] + ADP[c] => pyruvate[c] + ATP[c] |
| r13 | transport of water | H_2_O[c] <=> H_2_O[e] |
| r14 | pyruvate decarboxylate | pyruvate[c] + NAD[c] => acetylCoA[c] + formate[c] + NADH[c] |
| r15 | tca reaction 1 | acetylCoA[c] + oxaloacetate[c] => citrate[c] |
| r16 | tca reaction 2 | citrate[c] => isocitrate[c] |
| r17 | tca reaction 3 | isocitrate[c] => succinate[c] + glyoxylate[c] |
| r18 | tca reaction 4 | oxaloacetate[c] + NADH[c] => malate[c] + NAD[c] |
| r19 | tca reaction 5 | malate[c] => fumarate[c] |
| r20 | tca reaction 6 | fumarate[c] + NADH[c] => succinate[c] + NAD[c] |
| r21 | tca reaction 7 | glyoxylate[c] + acetylCoA[c] + H_2_O[c] => malate[c] |
| r22 | transport of succinate | succinate[c] => succinate[e] |
| r23 | transport of CO_2_ | CO_2_[e] => CO_2_[c] |
| r24 | ppp reaction 1 | xylulose 5-phosphate[c] <=> ribulose 5-phosphate[c] |
| r25 | ppp reaction 2 | ribulose 5-phosphate[c] <=> ribose 5-phosphate[c] |
| r26 | ppp reaction 3 | xylulose 5-phosphate[c] + ribose 5-phosphate[c] <=> sedoheptulose 7-phosphate[c] + glyceraldehyde 3-phosphate[c] |
| r27 | ppp reaction 4 | sedoheptulose 7-phosphate[c] + glyceraldehyde 3-phosphate[c] <=> erythrose 4-phosphate[c] + fructose 6-phosphate[c] |
| r28 | ppp reaction 5 | erythrose 4-phosphate[c] + xylulose 5-phosphate[c] <=> glyceraldehyde 3-phosphate[c] + fructose 6-phosphate[c] |
| r29 | tca reaction 8 | PEP[c] + CO_2_[c] => oxaloacetate[c] + phosphate[c] |
| r30 | phosphate transport | phosphate[e] => phosphate[c] |
| r31 | ADP transport | ADP[e] <=> ADP[c] |
| r32 | transport of formate | formate[c] <=> formate[e] |
| exc1 | uptake of glucose | glucose[b] => glucose[e] |
| exc2 | production or uptake of water | H_2_O[e] <=> H_2_O[b] |
| exc3 | production or uptake of CO_2_ | CO_2_[b] => CO_2_[e] |
| exc4 | production of succinate | succinate[e] => succinate[b] |
| exc5 | uptake of xylose | D-xylose[b] => D-xylose[e] |
| exc6 | production or uptake of P | phosphate[b] <=> phosphate[e] |
| exc7 | production or uptake of ADP | ADP[e] <=> ADP[b] |
| exc8 | production or uptake of formate | formate[b] <=> formate[e] |
| fake1 | hydrolysis of ATP | ATP[c] + H_2_O[c] <=> phosphate[c] + ADP[c] |

**S1 Table. The isolated metabolic subnet model of YL104H.**

The information about reversibility or irreversibility of included reactions was extracted from KEGG database (http://www.kegg.com).
